# Uptake of Ranibizumab but Not Bevacizumab into Uveal Melanoma Cells Correlates with a Sustained Decline in VEGF-A Levels and Metastatic Activities

**DOI:** 10.3390/cancers11060868

**Published:** 2019-06-21

**Authors:** Aysegül Tura, Vera E. Pawlik, Martin Rudolf, Justus S. Ernesti, Jan-Niklas Stutzer, Salvatore Grisanti, Mahdy Ranjbar

**Affiliations:** 1 Department of Ophthalmology, University of Lübeck, Ratzeburger Allee 160, 23538 Lübeck, Germany; ayseguel.tura@uksh.de (A.T.); vera.pawlik@uksh.de (V.E.P.); salvatore.grisanti@uksh.de (S.G.); 2 Laboratory for Angiogenesis & Ocular Cell Transplantation, University of Lübeck, Ratzeburger Allee 160, 23538 Lübeck, Germany; mirudolf@aol.com (M.R.); justus-satoshi.ernesti@uksh.de (J.S.E.); janniklas.stutzer@uksh.de (J.-N.S.)

**Keywords:** uveal melanoma, VEGF-A, angiogenesis, bevacizumab, ranibizumab

## Abstract

Despite the implication of vascular endothelial growth factor-A (VEGF-A) in the pathophysiology of uveal melanoma (UM), the anti-VEGF-A antibody bevacizumab yielded conflicting results on UM growth. Here, we evaluated whether bevacizumab and ranibizumab, a humanized Fab-fragment against VEGF-A, can enter UM cells and induce a sustained physiological response. The primary and metastatic UM cell lines Mel-270 and OMM-2.5 were exposed to bevacizumab or ranibizumab for one day and were maintained further in untreated medium for a total of three days. Both antibodies significantly reduced the levels of extracellular VEGF-A and the angiogenic potential of the conditioned medium after one day. These inhibitory effects of bevacizumab diminished by day three. Ranibizumab suppressed the metabolic activity, proliferation, and intracellular VEGF-A levels in a cell-type and concentration-dependent manner, whereas bevacizumab exerted no effect. Both drugs were detected inside early endosomes within the UM cells, with the stronger and sustained colocalization of ranibizumab. Our results therefore demonstrated the more potent and persistent suppressive activity of ranibizumab on the UM cells, possibly due to its higher level of uptake and prolonged intracellular retention. Further research on the endosome dynamics in UM cells might provide valuable insight into the response of these heterogenous tumors to therapeutic antibodies.

## 1. Introduction

Uveal melanoma (UM) is the most common primary intraocular malignancy arising in adults. Current treatments of UM depend on several clinical factors and include radiotherapy, enucleation, transpupillary thermotherapy, as well as local resection [1]. Despite tremendous progress in the diagnosis and local control of the primary tumor, up to 50% of UM patients may develop systemic metastases, leading to their currently inevitable death from this disease [2].

Since metastases of UM occur generally through the hematogenous route, dissemination of the primary tumor cells into peripheral blood is a prerequisite [3]. Accordingly, angiogenesis is a hallmark of tumor growth and invasion in primary UM and its metastatic disease [4,5]. Vascular endothelial growth factor A (VEGF-A) plays the central role in promoting angiogenesis by binding its specific tyrosine kinase VEGF receptors VEGF-R1 and VEGF-R2, which are primarily expressed on the surface of vascular endothelial cells [6,7]. In addition, VEGF-A can stimulate the proliferation, survival, migration, and invasion of various established cancer cells [6,8]. VEGF-A thereby plays a crucial role in the preparatory steps for distant metastases by not only stimulating the development and maintenance of a sufficient tumor vasculature but also directly activating the tumor cells [6,8,9].

VEGF-A is also implicated in the pathophysiology of UM in both experimental and clinical studies. For instance, higher serum concentrations of VEGF-A were correlated with a raised metastatic frequency and poor survival in a murine UM model [10]. The serum levels of VEGF-A were also increased in the UM patients with metastases [11]. Likewise, VEGF-A was elevated in the vitreous and anterior chamber fluid of eyes with UM, with the latter event being significantly correlated to the largest tumor diameter and tumor height [12,13]. The major sources of VEGF-A in the ocular fluids of UM patients are presumably the retina and the tumor cells, possibly due to the ischemic conditions in the tumor and the overlying, detached retina [13]. In addition, VEGF-A is involved in the radiation-induced vasculopathy in UM patients [14].

Anti-VEGF treatment with drugs like bevacizumab and ranibizumab has already proven very successful in other non-malignant, neovascular ocular diseases [15]. While bevacizumab is a full-length humanized antibody (149 kDa), ranibizumab is a smaller (48 kDa) humanized Fab-fragment [16]. We have previously demonstrated that both of these drugs do effectively inhibit extracellular VEGF-A secreted by retinal pigment epithelium (RPE), but differ considerably with regard to additional intracellular pathways related to the VEGF-A metabolism of RPE cells [17,18]. Since bevacizumab has been widely used for the treatment of radiation-induced vasculopathy, its potential to prevent the growth of residual UM cells has also gained considerable interest [14,19]. However, contrary to expectations, bevacizumab treatment yielded highly conflicting results on UM growth in experimental and clinical studies [5]. While some in vitro studies reported a considerable decline in the metastatic activities of UM cells in response to bevacizumab [19,20], other groups observed such effects in a cell-type dependent manner [21,22]. In mouse models of UM, bevacizumab could significantly prevent the growth and metastases of the melanoma cells that were inoculated into the choroid [20], but resulted in the paradoxical growth of a mouse melanoma cell line that was injected into the anterior chamber [19]. Bevacizumab also failed to suppress tumor progression in three UM patients who were erroneously diagnosed with choroidal neovascularization and have received multiple intravitreal injections of this antibody [23]. Moreover, the injection of bevacizumab into the vitreous of two UM patients with large tumors was succeeded by a 26–31% increase in tumor size within two weeks, which necessitated the premature termination of a clinical study [24]. Yet, the molecular mechanisms of these disconcerting results are still not well understood.

The efficacy of bevacizumab treatment is apparently influenced by the environmental context, where the interplay of UM cells with the endothelial and immune cells along with other extracellular factors in a distinct ocular compartment may be defining the final outcome. However, it is also not known, whether the individual UM cells, which usually exhibit morphological and genetic heterogeneity within a patient [25,26,27], differ in their ability to process such therapeutic antibodies intracellularly. In this study, we therefore aimed to evaluate this latter aspect using the well-characterized UM cell lines Mel-270 and OMM-2.5, which were derived from the primary tumor and liver metastases, respectively, of an UM patient [28,29]. Cells were incubated with bevacizumab or ranibizumab for one day and maintained further in untreated medium for a total of three days. Here, we report for the first time that both of these antibodies could be internalized into the UM cells, with the stronger and prolonged colocalization of ranibizumab into early endosomes. Ranibizumab could also suppress the metabolism, proliferation, VEGF-A levels, and angiogenic potential of UM cells more effectively than bevacizumab, possibly due to its longer intracellular retention. Our findings therefore provide the first evidence to the significance of endocytosis on the efficacy of therapeutic antibodies in UM cells.

## 2. Results

### 2.1. Metabolic Activity of Uveal Melanoma Cells Was Impaired by Ranibizumab but Not Bevacizumab

**Figure 1:**
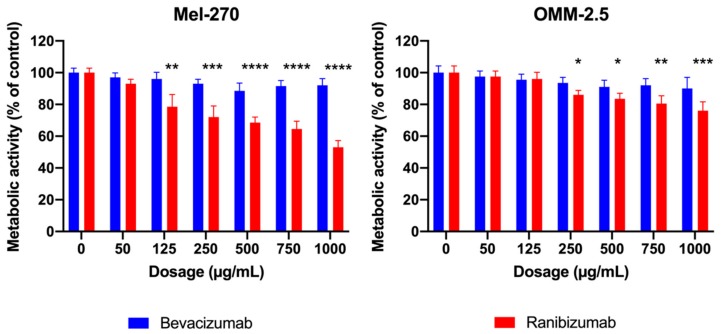
MTT assay demonstrating the metabolic activity of Mel-270 and OMM-2.5 cells after incubation with varying doses of bevacizumab and ranibizumab for 1 day. Bevacizumab did not show any significant impact on metabolic activity in either cell type. Ranibizumab exerted a dose- and cell-type-dependent effect, with 125 µg/mL being sufficient to reduce the metbolism of Mel-270 cultures, whereas at least 250 µg/mL were required to impair the OMM-2.5 cells. * *p* < 0.05, ** *p* < 0.01, *** *p* < 0.001, **** *p* < 0.0001.

After subjecting the Mel-270 and OMM-2.5 cultures to various dosages (50–1000 µg/mL) of bevacizumab or ranibizumab for one day, metabolic activity was determined by the MTT assay (Figure 1). None of the tested dosages of bevacizumab affected the metabolic activity of these cells, whereas ranibizumab exerted a cell-type and dose-dependent effect. While ranibizumab as low as 125 µg/mL led to a significant decrease of 21.5% in the metabolism of Mel-270 cells, a concentration of at least 250 µg/mL was needed to reduce the metabolic activity of OMM-2.5 cells significantly by 14%. Henceforth we decided to use both of these dosages for further experiments.

### 2.2. Ranibizumab Had a Longer Lasting and Stronger Impact on the VEGF-A Metabolism than Bevacizumab

UM cells were incubated with either bevacizumab or ranibizumab at a concentration of 125 µg/mL and 250 µg/mL for 1 day. The medium was then replaced daily with fresh medium that did not contain these antibodies and the cells were incubated further for a total of three days. The amount of extracellular and intracellular VEGF-A was quantified by ELISA and immunocytochemistry, respectively, after days one and three.

**Figure 2:**
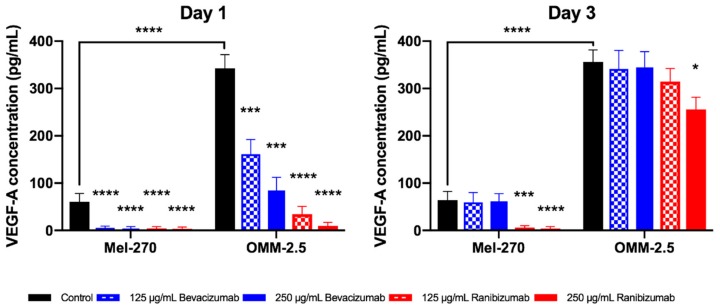
Extracellular vascular endothelial growth factor-A (VEGF-A) levels of Mel-270 und OMM-2.5 cells after a one-day exposure to bevacizumab or ranibizumab as determined by ELISA. Metastatic OMM-2.5 cells secreted significantly more VEGF-A than the corresponding primary tumor cells (Mel-270). Bevacizumab suppressed VEGF-A levels in the supernatant of both cells for a short period (one day). Ranibizumab at a concentration of 250 µg/mL was still able to significantly reduce the amount of VEGF-A in both cell types after three days. * *p* < 0.05, *** *p* < 0.001, **** *p* < 0.0001.

The basal level of extracellular VEGF-A was approximately 5.5-fold higher in the untreated metastatic OMM-2.5 cells compared to the corresponding primary tumor cells (Mel-270) at both time points. After the first day, both concentrations of bevacizumab and ranibizumab led to a significant decrease in extracellular VEGF-A levels in Mel-270 as well as OMM-2.5 cells (Figure 2). At day three, the effect of bevacizumab disappeared, as the VEGF-A levels in the supernatants recovered and were not statistically different from the controls. In contrast, ranibizumab showed a cell- and dose-dependent effect at this time point: In Mel-270 cultures, extracellular VEGF-A was still suppressed by almost 95%, but in OMM-2.5 cells, VEGF-A levels partly improved and only the higher dosage of 250 µg/mL was able to result in a statistically significant reduction by approximately 30% compared to the untreated cells.

**Figure 3:**
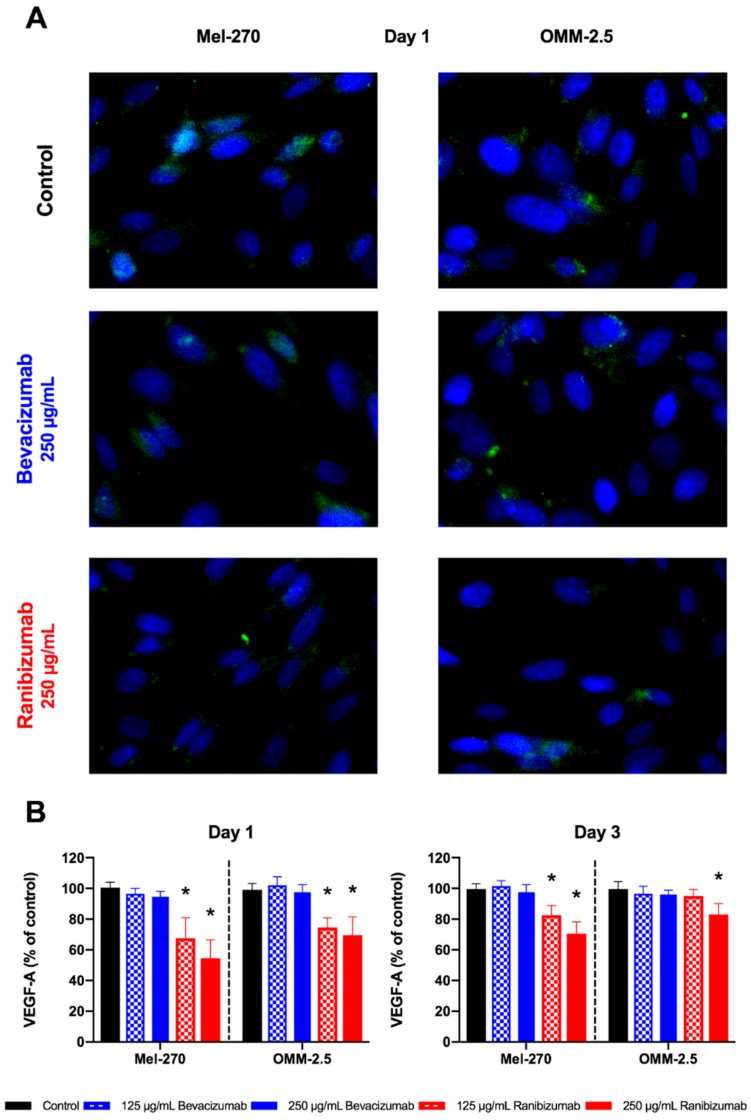
Intracellular VEGF-A levels after a one-day exposure to bevacizumab or ranibizumab. (**A**) Intracellular VEGF-A was evaluated by measuring the number of Alexa488-positive (green) particles. Cell nuclei were stained with DAPI (blue). Images were acquired at 400× magnification. (**B**) Bevacizumab did not reduce the intracellular VEGF-A levels in any cell type, dosage, or time-interval. Ranibizumab led to a statistically significant decrease of intracellular VEGF-A in both cells at day one. This significant effect persisted for both concentrations of ranibizumab in Mel-270 cultures at day three, but only for the 250 µg/mL dose in OMM-2.5 cells. * *p* < 0.05.

Intracellular VEGF-A levels showed an inconsistent reaction pattern (Figure 3). Bevacizumab did not significantly alter the amount of intracellular VEGF in neither Mel-270 nor OMM-2.5 cells, regardless of the applied concentration or the day of evaluation. Either dosage of ranibizumab significantly decreased the amount of VEGF-A within the Mel-270 and OMM-2.5 cells by 25–45% at day one. This effect was maintained further in Mel-270 cultures at day three, as VEGF-A levels persisted to be significantly lower in comparison to controls. In OMM-2.5 cells, the amount of intracellular VEGF-A normalized at day three for 125 µg/mL ranibizumab, but was still significantly reduced by 17% for the double dose.

### 2.3. More Ranibizumab than Bevacizumab Was Taken up into Uveal Melanoma Cells

**Figure 4:**
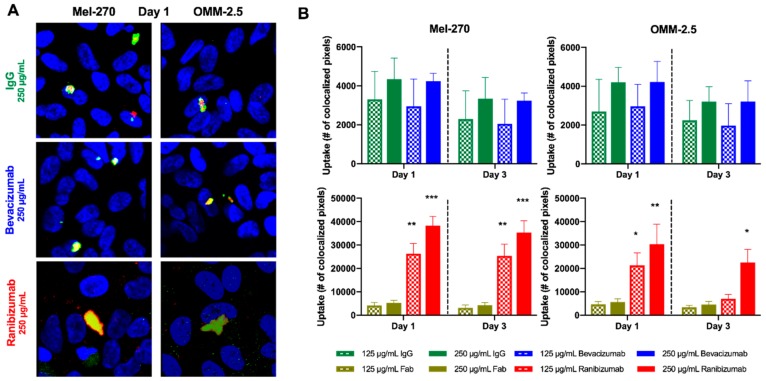
Intracellular uptake of bevacizumab and ranibizumab into UM cells after a one-day exposure to these antibodies. Cells were incubated with fluorescently labeled bevacizumab or ranibizumab for one day, then maintained in daily replaced untreated medium for up to three days. (**A**) Colocalization of the Dylight650-labeled drugs (red) with Alexa488-labeled, Rab5-positive endosomes (green) was evaluated by confocal microscopy. Cell nuclei were stained with DAPI (blue). Images were acquired at 400× magnification. (**B**) Considerable amounts of ranibizumab, but not bevacizumab were taken up into both Mel-270 and OMM-2.5 cells. While the accumulation of ranibizumab in the Mel-270 cultures persisted well for three days, its levels subsided in the OMM-2.5 cells after three days, with only the double dose of 250 µg/mL being detected at significantly higher levels compared to the isotype control. * *p* < 0.05, ** *p* < 0.01, *** *p* < 0.001.

Bevacizumab and ranibizumab were labeled with a fluorescent dye and then added to the UM cultures for one day to evaluate the uptake within the cells. Afterwards, the cells were maintained in fresh medium without antibodies that was replaced every 24 h for two further days. Cells were then processed for fluorescence-immunocytochemistry for the early endosomal marker Rab5. Colocalization of labeled bevacizumab or ranibizumab into early endosomes was evaluated by confocal microscopy (Figure 4).

Bevacizumab showed a moderate intracellular uptake into the UM cells at a rate similar to the equimolar isotype control of unspecific human IgG. This result was independent of the tested bevacizumab dosage and time of evaluation. By contrast, significant amounts of ranibizumab were taken up into the Mel-270 and OMM-2.5 cells in a dose-dependent manner, which exceeded the equimolar isotype control of unspecific human Fab-fragment by 5- up to 7-fold. In Mel-270 cells, the amount of ranibizumab did not decrease considerably from day one to day three, but there were still significant quantities of ranibizumab detectable at the latter time point. In OMM-2.5 cultures, ranibizumab molecules could remain significantly higher than the control at day three only when the double dose of 250 µg/mL was applied.

### 2.4. Cell Proliferation of Uveal Melanoma Cells Was Inhibited by Ranibizumab but Not Bevacizumab

**Figure 5:**
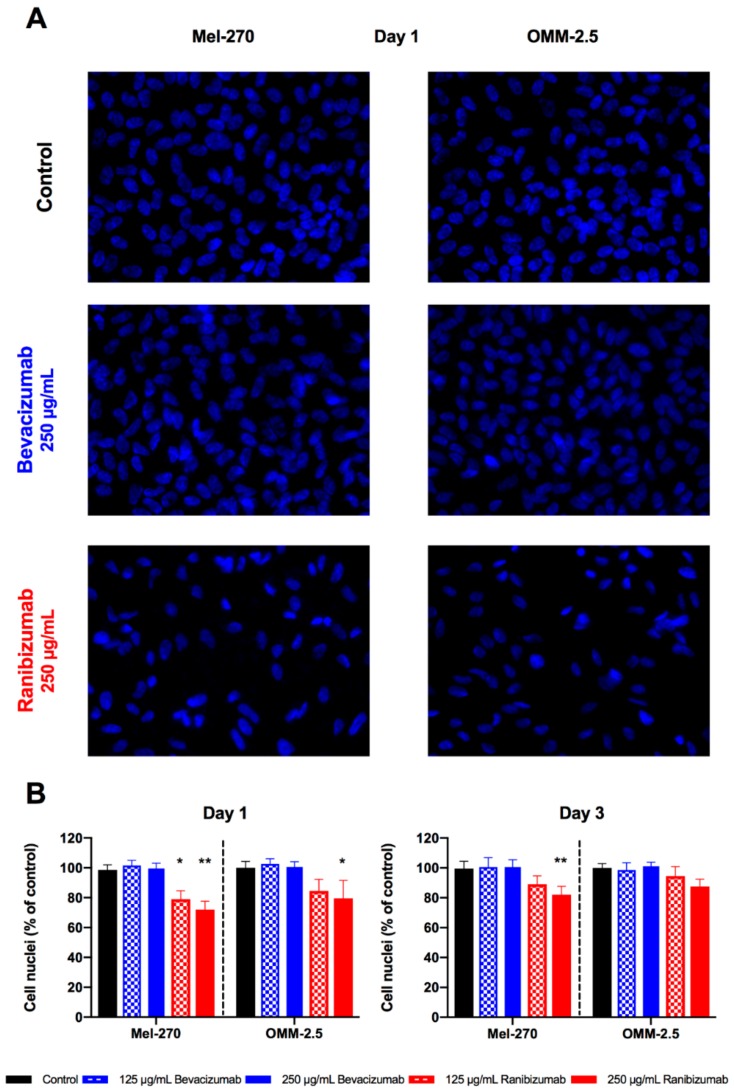
Effect of a 1-day exposure to bevacizumab and ranibizumab on uveal melanoma (UM) cell proliferation. (**A**) Fluorescent images were binarized and the number of cell nuclei were automatically counted. Images are shown for both UM cell lines after incubation with 250 µg/mL bevacizumab or ranibizumab at day one, compared to the untreated (control) cells. Images were acquired at 200× magnification. (**B**) Bevacizumab did not have any significant effect on cell proliferation, regardless of the examined circumstances. Proliferation of Mel-270 cultures were impaired by both dosages of ranibizumab at day one, but the double dose was required to maintain this effect by day three. In OMM-2.5 cultures, only the high dosage of ranibizumab could exert a significant anti-proliferative effect at day one, which no longer persisted at day three. * *p* < 0.05, ** *p* < 0.01.

The impact of bevacizumab and ranibizumab on UM proliferation was evaluated by counting the cell nuclei after exposure to these antibodies for one day and further incubation in antibody-free medium for two more days (Figure 5). Bevacizumab did not have a significant effect on the proliferation of either UM cell line, regardless of the applied concentration and the day of evaluation. Ranibizumab reduced the proliferation of Mel-270 cells by 20–30% at both concentrations on day one. At day three, the proliferation of these cells treated with 125 µg/mL of ranibizumab recuperated and no significant difference to untreated controls was detectable. However, the double dose led to a significant decrease in cell proliferation by 18%, even at day three. In OMM-2.5 cultures, ranibizumab induced a statistically considerable reduction by 20% only at day one, when given at a concentration of 250 µg/mL. This effect diminished below the level of significance by day three.

### 2.5. Ranibizumab Had a Stronger Anti-Angiogenic Impact than Bevacizumab in a Tube Formation Assay

**Figure 6:**
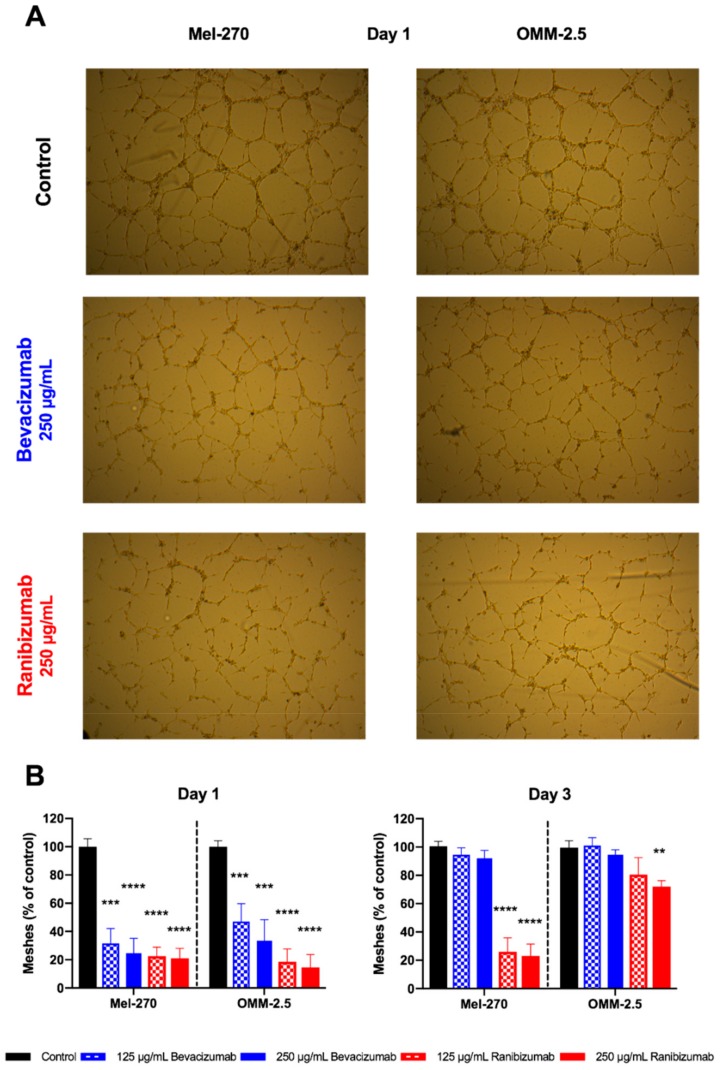
Angiogenic activity of UM cells after a one-day exposure to bevacizumab or ranibizumab. (**A**) Human umbilical vein endothelial cells (HUVECs) were seeded on matrigel and incubated with the conditioned medium of Mel-270 or OMM-2.5 cultures. The number of fully shaped meshes was automatically counted. Images are shown for day one conditioned medium from Mel-270 and OMM-2.5 cells after a one-day incubation with 250 µg/mL bevacizumab or ranibizumab, compared to the conditioned medium of untreated (control) cells. Images were acquired at 50× magnification. (**B**) Exposure of the UM cells to bevacizumab for one day significantly suppressed the pro-angiogenic impact of the supernatant. However, this effect was not maintained in the conditioned medium collected after three days. Ranibizumab was able to significantly weaken the angiogenic power of the Mel-270 culture supernatant at both time points. In OMM-2.5 cells, the persistence of this anti-angiogenic effect for three days was observed only when the double dose was applied. ** *p* < 0.01, *** *p* < 0.001, **** *p* < 0.0001.

The impact of a one-day exposure to bevacizumab and ranibizumab on the pro-angiogenic capacity of UM cells was evaluated by the common tube formation assay as the number of fully shaped meshes was considered (Figure 6). Bevacizumab in either concentration significantly weakened the pro-angiogenic power of the Mel-270 and OMM-2.5 supernatants by 50–75%, respectively, at day one. However, this anti-angiogenic effect was completely gone and no difference to untreated controls was existent at day three. Both dosages of ranibizumab reduced the pro-angiogenic impact of the Mel-270 supernatant by 75–80% at each time point. With regard to the OMM-2.5 conditioned medium, either dose of ranibizumab was anti-angiogenic at day one, with an 80–85% decline in the number of full meshes, but only the double dose was still able to weaken the pro-angiogenic ability of the supernatant by 30% at day three.

## 3. Discussion

Despite the evidence that supports the role of VEGF-A-mediated angiogenesis in the growth and metastases of UM cells, application of the anti-VEGF-A antibody bevacizumab has so far yielded contradictory outcomes in both experimental and clinical settings. These perplexing results were mainly attributed to the possible impact of bevacizumab on the anti-angiogenic splice variants of VEGF-A or the development of resistance in UM cells, which remain to be clarified in more detail [5,19,24]. However, it is still not known, whether the therapeutic anti-VEGF-A antibodies can be taken up into the UM cells and induce a sustained physiological response, which would influence their efficacy. In this study, we initially exposed the UM cell lines Mel-270 and OMM-2.5 to bevacizumab or ranibizumab for one day and determined the optimal concentration range by a metabolic activity assay. We then evaluated the temporal outcomes of a short-term (one-day) exposure to these antibodies on the extent of drug uptake, VEGF-A levels, proliferation, and angiogenic activity. Incubation with bevacizumab or ranibizumab significantly reduced the levels of extracellular VEGF-A and the angiogenic potential of the conditioned medium of UM cells after one day. However, these inhibitory effects of bevacizumab subsided by day three. Bevacizumab also failed to suppress the levels of intracellular VEGF-A or the metabolic activity and proliferation of UM cells at any time point or concentration. In contrast, ranibizumab could significantly interfere with these events in a cell-type and concentration-dependent manner.

The inhibitory effect of ranibizumab was more prevalent on the Mel-270 cells compared to the OMM-2.5 cells, possibly due to the 5.5-fold lower basal levels of extracellular VEGF-A in the conditioned medium of the former cell type. Incubation of the Mel-270 cells with ranibizumab at the clinically relevant dose of 125 µg/mL led to a sustained reduction in the total levels of VEGF-A and the angiogenic activity after three days, as well as an approximately 20% decrease in the metabolism and proliferation after one day. However, the anti-proliferative effect after three days could only be sustained in response to the double dosage of 250 µg/mL. In OMM-2.5 cells, this double dosage was required to maintain the VEGF-A downregulation and anti-angiogenic activity after three days as well as the suppression of metabolic activity and proliferation after one day. The anti-proliferative effect of ranibizumab on the OMM-2.5 cells diminished after three days even at this increased dose. These findings therefore indicate a higher basal level of metabolic activity and resistance in the OMM-2.5 cells, which are of metastatic origin, as opposed to the Mel-270 cells, which were derived from the primary tumor of the same patient [28,29]. Since the primary UM samples tend to exhibit heterogeneity within themselves [25,26,27], these findings also underscore the necessity to evaluate multiple UM cell lines to gain a more comprehensive insight into the efficacy of treatments.

A possible reason accounting for the lower efficacy of bevacizumab versus ranibizumab might be the molar difference of these antibodies. As stated earlier, bevacizumab is a full-length, humanized, bivalent antibody (MW: 149 kDa), whereas ranibizumab is a recombinant, monovalent Fab-fragment (MW: 48 kDa) [15,16,30,31,32]. The concentrations of 125 µg/mL and 250 µg/mL would therefore equal to 0.84 and 1.68 mM of bevacizumab, respectively, which are approximately one third of the molarity of ranibizumab (2.6 mM and 5.20 mM, respectively) used in our study. However, the bivalence of bevacizumab would double the amount of available VEGF-binding sites [32]. Therefore, the administration of 250 µg/mL (1.68 mM) bevacizumab would introduce 3.36 mM of VEGF-binding domains into the culture environment, whereas for 125 µg/mL (2.60 mM) ranibizumab, the available epitope binding sites would be 2.60 mM. Since the treatment of UM cells with 125 µg/mL of ranibizumab could suppress the metabolism, proliferation, VEGF-A levels, and angiogenic activity more potently than 250 µg/mL bevacizumab, the molar differences do not appear to be the sole factor influencing the efficacy of these antibodies.

A further reason underlying the disparity in the early and prolonged effects of bevacizumab versus ranibizumab might be attributed to the uptake levels of these drugs. In this study, we provide the first evidence that both of these drugs were internalized into the UM cells, being colocalized with the early endosome marker Rab5 by confocal microscopy. Bevacizumab was detected inside the Mel-270 and OMM-2.5 cells at a similar range to the equimolar isotype control of unspecific human IgG at both time points. In contrast, the uptake level of ranibizumab exceeded its isotype control and bevacizumab by approximately 6- up to 7-fold after one day. These high levels were maintained for three days in the Mel-270 cells but declined by around 60% in the OMM-2.5 cells treated with the lower dose (125 µg/mL) of ranibizumab, possibly due to the higher metabolic activity and endosome turnover rate of the latter cell type.

We have previously reported that the uptake of bevacizumab into RPE cells is highly dependent on the Fc receptor [17]. Since the Fab-fragment ranibizumab lacks an Fc region [30], its entry into the cells must be mediated by alternative mechanisms. The higher uptake level of ranibizumab compared to its isotype control suggests that the specific VEGF-binding site of ranibizumab might have facilitated its internalization. We have indeed previously demonstrated that blocking the VEGF-R2 could significantly impair the accumulation of ranibizumab in the early endosomes of RPE cells, suggesting that ranibizumab may be undergoing endocytosis together with the VEGF/VEGF-R2 complex [18]. It would be therefore very interesting to perform further studies on the possible involvement of VEGF-R2 in the endosome-mediated transport of ranibizumab into the UM cells.

Receptor-mediated endocytosis is indeed a critical event which determines the fate of internalized cell surface proteins, directing them either into the degradation pathways in lysosomes or promoting their recycling back to the plasma membrane. Endosomes which contain activated receptors can also influence the duration of the signaling, with their small volume that facilitates protein-protein interactions and their connections to the microtubular networks that enable the transport to different cellular compartments. Moreover, the nature of the endosomal cargo itself can affect the fate of the endosomes. Accordingly, endosome dynamics belong to the pathways that are dysregulated in various cancer cells [33,34,35,36,37]. However, the significance of endocytosis on the drug efficacy in UM cells has not received attention so far. In our study, the short-term application of ranibizumab on to UM cells resulted in a more potent and longer-term reduction in the total VEGF-A levels, cell proliferation, and angiogenic activity compared to bevacizumab. This effect was highly related to the amount of drug retention inside the cells over longer periods, with the stronger and sustained colocalization of ranibizumab into early endosomes after three days. Our findings therefore provide the first evidence to the involvement of endosomes in regulating the long-term actions of internalized anti-VEGF-A antibodies in UM cells. As a next step, it would be very interesting to trace the fates of the endosomes that carry bevacizumab versus ranibizumab in order to gain more insight into the prolonged mechanisms of action induced by these treatments.

Interestingly, the genes encoding the early endosomal proteins Rab5 (NCBI Gene ID: 5868) and APPL1 (Gene ID: 26060) are localized to chromosome 3p24.3 and 3p14.3, respectively. Likewise, the late endosomal protein Rab7, which mainly directs the endosomal cargo towards the lysosomal degradation pathway [35,36], is encoded by a gene on chromosome 3q21.3 (Gene ID: 7879). Moreover, the genes for the transferrin protein (Gene ID: 7018) and its receptor (Gene ID: 7037), which play central roles in the recycling of endosomal contents on to plasma membrane [35], reside on chromosome 3q22.1 and 3q29, respectively. The loss of one copy of chromosome-3 (Monosomy-3) is indeed the most important prognostic factor in UM, which is significantly associated with a higher risk of metastases [38,39]. Partial deletions of 3p or 3q were also observed in the UM samples with a less favorable prognosis [39]. However, there is to our knowledge no information on the correlation of chromosome-3 abnormalities with the endosome function in UM cells. Remarkably, the Mel-270 cells were found to be harboring the loss of 3p24 and 3q21.2-3q24 loci [28], which contain the Rab5 and Rab7 genes, respectively. Monosomy-3 was also detected in approximately 50% of the primary tumor cells of the patient, from whom the Mel-270 and OMM-2.5 cells were generated [28,29]. Although the cell lines that were maintained in culture over long periods can acquire genotypic differences, which can make them diverge significantly from the cells of the original tissue that they were derived from, the cell lines used in our study are nevertheless very likely to have aberrant endosomal dynamics. To evaluate the possible contribution of this factor to the efficacy of drug therapy in UM cells, it would be very interesting to perform further investigations on the outcomes of bevacizumab and ranibizumab treatment with regard to the endosome fate and Monosomy-3 status.

It also remains to be determined, whether the Fc receptor is involved in the internalization of bevacizumab into the UM cells. Notably, the genes encoding the majority of the known members of the Fc receptor family and Fc receptor-like molecules are clustered on chromosome 1 [40,41,42]. UM cells also exhibit clinically relevant aberrations on this chromosome, with the loss of 1p or the gain of 1q being more frequently encountered in the metastasizing tumors [39,43]. However, it is not known yet, whether such anomalies would alter the Fc receptor profile of UM cells and influence the responsiveness to full length therapeutic antibodies such as bevacizumab. In our study, we did not analyze the presence of these aberrations in the Mel-270 or OMM-2.5 cells. To our knowledge, these cells have not yet been characterized for the alterations on chromosome 1 in the literature, either. Further analysis on the association of chromosome 1 aberrations with the Fc receptor profile of UM cells and its impact on the intracellular fate of bevacizumab might therefore provide novel insight into the mechanisms that underlie the contradictory actions of bevacizumab on UM growth reported so far.

A recent study has reported that the anti-VEGF drugs such as aflibercept, bevacizumab, and ranibizumab can compete with the anti-VEGF antibodies in commercially available ELISA kits for binding VEGF, resulting in the underestimation of VEGF-A concentration by 2- to 100-fold [44]. This effect can happen when the antibodies used for VEGF-A detection occupy the receptor binding domain of VEGF-A, which is also the target site of ranibizumab and bevacizumab. The receptor binding domain of human VEGF-A is encoded by the exons 1–4 of the VEGF-A gene [45,46]. For the intracellular detection of VEGF-A in our study, we used a polyclonal rabbit anti-human VEGF-A antibody (Life Technologies /Thermo Fisher PA1-16948), that was raised against a synthetic peptide between the residues 180–232 of the human VEGF-A protein sequence. Since these residues are localized to the exon 8 of VEGF-A gene [46], this antibody would not be expected to recognize the receptor binding domain of VEGF-A. In other words, the treatment with ranibizumab or bevacizumab would not have been likely to interfere with the intracellular detection of VEGF-A in our study.

Since the binding site of the VEGF-A antibody in our ELISA-Kit was not disclosed and this kit was recently reported to be susceptible to an interference with ranibizumab and bevacizumab [44], we cannot exclude the possibility of an underestimation of extracellular VEGF-A concentration in response to ranibizumab and bevacizumab in our study. However, our study design and the differing results for the Mel-270 and OMM-2.5 cells nevertheless suggest significant physiological responses. Both cell lines were exposed to ranibizumab or bevacizumab for one day, followed by daily rinsing and replenishing with untreated medium for a total of three days. This treatment would substantially deplete the extracellular ranibizumab or bevacizumab that was introduced on the first day. Though we cannot rule out the possible release of intracellular ranibizumab or bevacizumab back into the fresh culture medium on day three, the concentration of these drugs would also be considerably lower than the original dose introduced on day one. Since the extracellular levels of VEGF-A decreased by 95% in the Mel-270 cells after both the first and third days, this effect appears to be independent of the extracellular ranibizumab that might have interfered with the VEGF detection. Our tube formation assay also demonstrated the long-term inhibitory effect of the transient exposure to ranibizumab, but not bevacizumab on the angiogenic potential of the UM cells, providing further support to the significant differences in the extracellular VEGF-A levels induced by these treatments.

Our results on the efficacy of bevacizumab and ranibizumab on UM cells were also highly consistent with previous reports. For instance, the anti-proliferative effect of bevacizumab could become significant at the concentrations above 2 mg/mL in two other studies, whereas the 250 µg/mL dose, which was introduced into the culture medium with 5–10% serum, had no effect [19,22]. Yet, in another study, incubation of the UM cell line 92.1 with 100 µg/mL bevacizumab for one day in serum-free medium could suppress the proliferation significantly by 20%, suggesting that certain serum factors may be interfering with the bevacizumab activity [21]. Our findings demonstrating the stronger activity of ranibizumab at the double dose of 250 µg/mL were also supported by an earlier work on ciliary body melanoma cultures, which required this dosage for a significant decline in survival [47], and a very recent study on the proliferation of 92.1 cells, which could not be inhibited by 125 µg/mL ranibizumab [48]. Interestingly, the incubation of six different UM cultures with high concentrations of bevacizumab (above 2 mg/mL) under hypoxia resulted in the striking upregulation of VEGF mRNA levels in four of these cell lines, suggesting that an excessive VEGF inhibition in a hypoxic environment might activate the pro-angiogenic signaling through alternative pathways, which may be one of the reasons underlying the paradoxical effects of bevacizumab [19]. Since our analyses were performed under normoxic conditions, it would be very interesting to repeat our assays under hypoxia to gain more insight into the response of UM cells to VEGF inhibition in a more challenging environment.

Our findings are also supported by the previous studies on the pharmacokinetics of bevacizumab and ranibizumab in the eye. The former antibody had a longer elimination half-life of approximately 10 days in the aqueous humor of patients, compared to the three-day half-life of ranibizumab in primate eyes [32,49]. Bevacizumab also had a longer half-life in the vitreous of rabbits compared to ranibizumab [50], which was attributed to the protective effect of the Fc receptor [32]. However, it is to our knowledge not analyzed yet, how much of the pharmacokinetic differences in such extracellular environments solely reflect the degradation or extraocular transport of these antibodies, rather than their possible intracellular uptake into the ocular cells. Interestingly, ranibizumab could be detected in the retina and to a lesser extent in the RPE of monkeys after intravitreal injection [51], suggesting the transport of this antibody through these tissues. We have also previously reported that the transient exposure of RPE cells to ranibizumab led to the intracellular uptake of this antibody, which was associated with a long-term suppressive effect on VEGF-A levels [18]. These results, together with our recent findings on UM cells, therefore support the view that, even though the therapeutic VEGF-A antibodies may be cleared from the extracellular compartments of the eye, their retention inside the cells may lead to sustained effects, which deserves further consideration.

Finally, we have to address one limitation of our study, as vehicle differences of ranibizumab and bevacizumab have not been considered, especially with regard to the control. This potential issue has also not been considered in previous ophthalmological studies that evaluated the efficacy of both drugs either individually or compared to each other. However, the vehicles of ranibizumab and bevacizumab do differ in their formulations. Ranibizumab is supplied as a 10 mg/mL solution in 10% trehalose dehydrate, 0.01% polysorbate 20 (Tween 20), and 10 mM (1.98 mg/mL) L-histidine in water for injection at pH 5.5. Bevacizumab is available at a concentration of 25 mg/mL in 6% trehalose dehydrate, 0.04% polysorbate 20 (Tween 20), 0.58% sodium phosphate (mono basic, monohydrate), and 0.12% sodium phosphate (dibasic, anhydrous) in water at pH 6.2. The major component that differs in the formulation of these vehicles is the amino acid L-histidine, which is already present in the RPMI-1640 medium at 96.7 µM. Though we could not find information on the concentration of histidine in fetal bovine serum (FBS), the levels of this free amino acid in the serum of normal men was approximately 1.183 mg/100 mL (76.3 µM). We therefore estimate that the supplementation of 5% FBS (as in our “light-medium”) would result in a minor increase of 3.8 µM, resulting in a total histidine level of approximately 100 µM in our basal medium. Addition of ranibizumab into this medium at the doses of 125 µg/mL (1/80 dilution) or 250 µg/mL (1/40 dilution) would introduce further L-histidine at 125 and 250 µM, respectively. We can therefore not rule out, whether the differences in L-histidine concentrations contributed to the stronger anti-carcinogenic activity of ranibizumab versus bevacizumab treatment in our study. Yet, a very recent study has reported that dietary histidine could indeed promote rather than inhibit the growth of central nervous system dedifferentiation tumors in a Myc-dependent pattern in a Drosophila model. Though we do not know, whether free histidine can alter the metabolism of UM cells, we consider it very interesting to consider this aspect and analyze its possible contribution to the treatment effects in forthcoming studies.

## 4. Materials and Methods

### 4.1. Cell Lines and Culturing Conditions

The human primary UM cell line Mel-270 and the corresponding metastatic UM cell line OMM-2.5 were kindly provided by Martine J. Jager (Leiden University Medical Center, Leiden, The Netherlands). Both the primary and metastatic UM cells were routinely grown under normoxic conditions at 37 °C in RPMI 1640 medium with 2 mM L-glutamine (Life Technologies, Darmstadt, Germany) supplemented with 10% fetal bovine serum (FBS; Biochrom, Berlin, Germany) and 1% penicillin/streptomycin (Biochrom), henceforth referred to in this article as “full medium”. Cells were passaged twice weekly and passages 9–14 were used for the experiments.

### 4.2. Metabolic Cell Activity

UM cells (5.000 cells per well) were seeded on 96-well plates and were allowed to grow for 24 h in full medium. Cells were then incubated with different concentrations (50–1000 µg/mL) of bevacizumab (Avastin^®^; Roche, Basel, Switzerland) or ranibizumab (Lucentis^®^; Novartis, Basel, Switzerland) diluted in RPMI 1640 medium with 2 mM L-glutamine supplemented with 5% FBS and 1% penicillin/streptomycin, henceforth referred to in this article as “light medium”. Controls were performed using drug-free light medium. After 24 h, 3-(4,5-dimethylthiazol-2-yl)-2,5-diphenyltetrazolium bromide (MTT) assay was performed. A 5 mg/mL stock solution of MTT in phosphate buffered saline (PBS) was added to the medium of each well at a final dilution factor of 1/10 and incubated for 3 h at 37 °C. Produced formazan was solubilized by dimethyl sulfoxide (DMSO) and quantified by light absorption at 570 nm with spectrophotometry (SpectraMax i3x, Molecular Devices GmbH, Biberach an der Riss, Germany).

### 4.3. VEGF-A Metabolism

UM cells (10.000 cells per well) were seeded on 8-well chamber slides (Nunc, Wiesbaden, Germany) and were allowed to grow for 24 h in full medium. Culture medium was then changed and cells were incubated further for 24 h with bevacizumab or ranibizumab, which were diluted in light medium to the final concentrations of 125 µg/mL and 250 µg/mL. Drug-free light medium was used as control. The supernatant was removed (Day 1) and stored at −80 °C before cells were washed and incubated with regular light medium. After 24 h, the medium was once again replaced by fresh light medium. After this final 24 h period, the supernatant was removed (Day 3) and stored at −80 °C before cells were washed with PBS and fixed in 4% paraformaldehyde-PBS. Samples were permeabilized and blocked in 0.1% Triton/5% goat serum in PBS for 2 h at room temperature (RT), and incubated with a rabbit-anti-human VEGF-A antibody (1:200; Life Technologies) overnight at 4 °C. After another wash with PBS, samples were incubated with a mixture of Alexa488-conjugated-mouse-anti-rabbit fluorescent secondary antibody (λ_ex_/λ_em_ = 495 nm/519 nm; 1:150; Merck Millipore, Darmstadt, Germany) for 1 h and 4′,6-diamidino-2-phenylindole (DAPI; λ_ex_/λ_em_ = 358 nm/461 nm; 1 µg/mL; Life Technologies) for 10 min at RT. Chambers were then removed and the slides were mounted using Mowiol (Sigma Aldrich, Steinheim, Germany). Stainings were visualized under an inverse fluorescence microscope (Leica DMI6000 B, Leica Microsystems, Wetzlar, Germany).

To assess the intracellular amount of VEGF-A, three randomly located images (200 × 300 µm^2^) were taken of each sample and were analyzed by semi-quantitative measurement of the number of Alexa488 (VEGF-A)-positive particles around DAPI-positive cell nuclei, using the ImageJ software (NIH, Bethesda, USA). The number of particles was normalized to the number of cell nuclei in each image.

To evaluate the quantity of extracellular VEGF-A, supernatants (Day 1 and Day 3) were analyzed for all isoforms by enzyme-linked immunosorbent assay (ELISA; R&D Systems, Minneapolis, MN, USA) using a multimode plate reader (SpectraMax M4), according to the manufacturer’s protocol.

### 4.4. Drug Uptake

UM cells (10.000 cells per well) were seeded on 8-well chamber slides (Nunc, Wiesbaden, Germany) and processed as described above. However, drugs were this time labeled with Dylight650 (λ_ex_/λ_em_ = 652 nm/672 nm; Life Technologies) according to the manufacturer’s protocol. Corresponding dosages of unspecific human IgG (Life Technologies) and Fab-fragment (Abcam, Berlin, Germany) were used as control. After fixation, samples were permeabilized, blocked, and incubated with a rabbit-anti-human Rab5 (early endosome marker) antibody (1:200; Life Technologies) overnight at 4 °C. Primary antibody binding was detected using an Alexa488-conjugated-mouse-anti-rabbit fluorescent secondary antibody (λ_ex_/λ_em_ = 495 nm/519 nm; 1:150; Merck Millipore) for 1 h. Cell nuclei were counterstained with DAPI and after removal of the chambers, slides were mounted using Mowiol. Stainings were assessed with a confocal fluorescence microscope (LSM 510 META, Carl Zeiss, Jena, Germany).

To evaluate drug uptake, three randomly located confocal fluorescence images (200 × 300 µm^2^) were taken of each sample and they were analyzed semi-quantitatively with regard to the colocalized Dylight650/Alexa488 (drug/Rab5) particles using ImageJ as previously reported [17,18].

### 4.5. Cell Proliferation

All slides from drug uptake experiments were also assessed with an inverse fluorescence microscope (Leica DMI6000 B). Three randomly located images (200 × 300 µm^2^) were taken of each sample. Images were exported to ImageJ and binarized to automatically count the cell nuclei for proliferation analyses.

### 4.6. Tube Formation Assay

HUVECs (ATCC, Manassas, VA, USA) were routinely cultured under normoxic conditions at 37 °C in F-12K medium (ATCC) supplemented with 10% FBS, 1% penicillin/streptomycin, 0.1% heparin (Sigma-Aldrich, Seelze, Germany) and 0.1% endothelial cell growth supplement (ECGS; Sigma-Aldrich). Cells were passaged twice weekly and passages 18–20 were used for the experiments.

Experiments were performed on µ-slides (Ibidi, Martinsried Germany) by placing 10 mL of Matrigel (Becton Dickinson, Heidelberg, Germany) diluted 1:1 in F-12K into the inner well of the slide. After gelation at 37 °C for 30 min, 10.000 HUVECs per well were seeded and cultured in full HUVEC medium for 2 h. Then the medium was replaced by the conditioned supernatant medium (Day 1 or Day 3), which was mixed with fresh HUVEC culture medium (1:1). Cells were incubated for another 10 h, before tubes formed and the structures were visualized by phase contrast (Leica DMI6000 B). Images were analyzed using the Angiogenesis Analyzer plugin for ImageJ (NIH) and the number of fully shaped meshes was taken into account for final evaluation.

### 4.7. Statistical Analysis

All experiments were conducted thrice, and in each experiment, data were generated from multiple replicates. Values are expressed as mean ± standard deviation. Statistics were performed with Prism 7 (GraphPad, La Jolla, San Diego, CA, USA) using a two-way analysis of variance (ANOVA) with Bonferroni correction. Results with *p* < 0.05 were considered significant.

## 5. Conclusions

In conclusion, our results demonstrated that ranibizumab can suppress the proliferation, metabolic activity, VEGF-A levels, and angiogenic potential of UM cells more effectively than bevacizumab, possibly due to the uptake efficiency and longer retention of the former antibody inside the cells. Therefore, these antibodies should not be simply regarded as external agents with a limited elimination half-life in ocular fluids, but their prolonged action inside the UM cells should be taken into consideration, as well. Our findings also provided the first evidence to the so far overlooked role of endosomes in modulating the long-term actions of anti-VEGF-A antibodies in UM cells, which deserves further investigation. Since the UM cells with Monosomy-3 are very likely to have abnormal endosome dynamics, analysis of additional UM cell lines which differ in the extent of Monosomy-3 would provide valuable insight into the response or resistance of these highly heterogenous tumors to therapeutic antibodies. Moreover, the characterization of the VEGF-A splice variants which are mainly affected by bevacizumab and ranibizumab might enable a more comprehensive understanding of the downstream effects of these interventions in UM cells. Further in vivo studies would also be indispensable to evaluate the efficacy of ranibizumab treatment on the suppression of UM growth and metastases in an environment where the tumor cells interact with the endothelial and immune cells.
